# Associations of attitudes towards electronic cigarettes with advertisement exposure and social determinants: a cross sectional study

**DOI:** 10.1186/s12971-017-0118-y

**Published:** 2017-02-13

**Authors:** Benjamin Reinhold, Rebecca Fischbein, Surya Sruthi Bhamidipalli, Jennifer Bryant, Deric R. Kenne

**Affiliations:** 1 College of Public Health, Kent State University, 800 E. Summit St, Kent, 44240 OH USA; 2 Health Policy and Management, College of Public Health, Kent State University, 800 E. Summit St, Kent, 44240 OH USA

**Keywords:** Electronic Cigarettes, Advertising, E-cigarettes, Nicotine, Perceptions, Harm, Transitional youth, Adolescents

## Abstract

**Background:**

The exposure of young adults to electronic cigarette (e-cigarette) advertisements has risen rapidly. E-cigarette ads have been shown to increase short term perceived acceptability of using e-cigarettes in places where traditional cigarettes are banned. We set out to investigate if advertising exposure was related to perceptions of harm, addictiveness, and acceptability of use of e-cigarettes in places where traditional cigarettes are banned.

**Methods:**

Using a cross-sectional design, 6037 students at a large Midwestern university between the ages of 18–24 were surveyed about e-cigarette use and smoking status. Bivariate analyses were performed associating perception of harm, addictiveness, and acceptability of e-cigarette use in places where smoking is banned with demographic and other background factors, and e-cigarette advertising exposure through different media channels. Logistic regression analyses were used to explore the relationship of these factors on perceptions of harm, addictiveness and acceptability of e-cigarette use in places where smoking is banned.

**Results:**

More than a quarter (27.4%) of respondents had used an e-cigarette, greater than half (53.2%) had seen an advertisement on TV and 42.0% had seen an advertisement on the Internet. Logistic regressions revealed that being white, male, an e-cigarette user, a smoker, having a mother who smoked, and Internet advertisement exposure were associated with lower perceived harm of e-cigarettes. The same factors, plus having seen advertisements on TV, were associated with increased likelihood of perceiving e-cigarette use in bars, stores, at work and in a dorm as acceptable. Perceiving use of e-cigarettes as acceptable in classrooms was also associated with the aforementioned factors and also included race. Only being male and an e-cigarette user were associated with lower perceived addictiveness of e-cigarettes.

**Conclusions:**

E-cigarette use is increasing in adolescents and young adults, as is exposure to e-cigarette advertising. Independent of nicotine use and demographics factors, e-cigarette advertising is associated with increased beliefs in acceptability of e-cigarette use in places where cigarettes are banned. E-cigarette advertisements may be responsible for normalizing e-cigarette use. Exposure to internet e-cigarette advertisements was associated with lower perceived harm; this may be due to the false health claims often made in internet advertisements.

## Background

Smoking is the leading cause of preventable death in the United States resulting in almost half a million deaths per year and an economic cost of $300 billion [1]. Nicotine is one of the most addictive commonly used drugs; only heroin and cocaine are more addictive [2]. Over the last 40 years efforts to reduce tobacco cigarette use among adolescents and young adults has been largely successful, during this time the proportion of 12^th^ graders (17–18 years of age) reporting daily tobacco cigarette use dropped from 27 to 6.7% [3]. However, new forms of nicotine use are gaining acceptance among youth. Electronic cigarettes (e-cigarettes) or Electronic Nicotine Delivery Systems (ENDS) are devices that heat a mixture of liquids (“e-juice”) that typically contain propylene glycol, glycerin and nicotine into an inhalable aerosol [4]. E-juice mixtures also often contain flavorings such as fruit, coffee or tobacco. E-cigarette use is growing rapidly among adolescents. In 2014, 13% of high school seniors reported having had a tobacco cigarette in the last 30 days, while 17% reported using an e-cigarette in that time frame [3]. Among 10^th^ graders (15–16 years of age) the disparity in use is more striking; 7 and 16% of 10^th^ graders have used tobacco cigarettes and e-cigarettes respectively in the last 30 days.

The emergence of e-cigarettes has led to concern among public health professionals that e-cigarettes may reverse the declines in tobacco use through nicotine addiction and potential renormalization of smoking among adolescents [5, 6]. Some claim e-cigarettes are effective smoking cessation tools and evidence suggests e-cigarettes may reduce number of tobacco cigarettes smoked [4]. However, conclusive evidence demonstrating e-cigarettes as aids in smoking cessation is lacking, and recent evidence suggests the use of e-cigarettes may actually reduce the likelihood of successfully quitting tobacco cigarettes [4]. In addition to the risk of providing a pathway to traditional cigarettes, current research suggests that some e-cigarette flavorings may be harmful when heated and inhaled. Specifically, prolonged inhalation of some flavorings contained in e-cigarettes could result in respiratory disease [7].

Until recently, regulation of electronic cigarettes fell to individual states, with several passing legislation that made electronic cigarettes subject to many of the regulations governing tobacco products (e.g., banning use in public buildings, restricting access to individuals at least 18 years of age). In May 2016, the Food and Drug Administration (FDA) published final rules that extended regulatory authority to include electronic cigarettes and other electronic nicotine delivery systems (ENDS) effective August 8, 2016. Under these new rules, e-cigarettes are subject to many of the same restrictions as conventional tobacco cigarettes, including requiring health warnings, pre-approval of products before market release, minimum age to purchase, and premarket review [8]. E-cigarette advertising and its effects on e-cigarette use and acceptance has not been studied extensively, but the limited available data are concerning.

A study of Nielsen data correlating television advertisement viewership with age revealed that young adults’ exposure to e-cigarette advertisements increased more than 300% between 2011 and 2013 [9]. While FDA restrictions prevent e-cigarette vendors from making claims about the efficacy of e-cigarettes for smoking cessation on television or the Internet, through third parties, sellers have exposed the public to Internet advertisements containing unsupported health and smoking cessation claims [10]. These advertisements appear to be quite effective with e-cigarette users who learned about e-cigarettes online demonstrating lower perceived risk of e-cigarettes than users who did not [11]. Adolescents seem particularly vulnerable to e-cigarette ads. Farrelly and colleagues found that exposure to just four e-cigarette ads increased by 50% the odds adolescents said they were going to try an e-cigarette soon, as well as increasing by 70% the odds that they would favor e-cigarette use in places where tobacco cigarettes are banned [12].

Given the potential impact of e-cigarette advertising on young adults, the current research examines exposure to e-cigarette advertising and its effect on e-cigarette attitudes and use among transitional age college students (ages 18–24). We surveyed young adults at a Midwestern University with the intent of answering the following questions: What are young adults’ perceptions of harm and acceptability of use of e-cigarettes? Does e-cigarette advertising have an effect on perception of harm and acceptability of use?

## Methods

The current study consisted of a cross-sectional sample of self-reported survey data collected at a large Midwestern University during the first week of February 2014. An online survey that included items about e-cigarette use, perceptions of harm and addictiveness of substances, acceptability of e-cigarette use in places where smoking is banned, and background information including smoking status was administered. The survey was distributed to all enrolled students (*N* = 35,299) at the university, and 9494 responded (response rate 27%). Students were informed that the survey was anonymous. In an effort to increase response rate, participation was incentivized with a prize drawing for several gift cards of varying denomination, a tablet computer, and headphones. Students were informed that entry into the prize drawing was optional and that contact information provided for the prize drawing could not be linked to individual survey responses. The design of the survey included skip patterns, prevented respondents from completing the survey more than once and from forwarding the survey to other individuals. Two email reminders were sent during the week the survey was active. The Institutional Review Board at Kent State University approved the study.

### Sample

While 9494 students ranging in age from 18 to 69 responded to the survey, this study includes only transitional age youth (18–24; *N* = 6819). Respondents who did not know if they had used an e-cigarette or did not know their smoking status were excluded from the analysis, which reduced the sample further to 6418. Because the original sample was disproportionate in terms of race and gender, population-based weighting was used to address potential non-response bias for these response classes. Consequently, participants who did not respond to one of the weighting class variables (gender, race, graduate/undergraduate status) were excluded from the analysis. This left data from 5983 respondents for the current study. Using the weighted data, the majority of respondents were female (61.3%) and white (79.8%). Nearly 20% of respondents reported as either a current or former smoker. Slightly over a quarter of students reported ever having used an e-cigarette (27.4%). Just under one third of participants had mothers who were either current or former smokers (32.7%).

### Measures

Demographic variables included age, gender, race, family income, respondent and parental tobacco smoking status and respondent lifetime e-cigarette use. Respondent smoking status was determined using the Brief Risk Factor Surveillance System (BRFSS) questionnaire criteria from the CDC (2009) [13]. Maternal smoking status was assessed with the question, “How would you classify your mother in terms of smoking cigarettes?”. Possible responses included “My mother is a regular smoker”, “My mother used to be a regular smoker, but has since quit”, and “My mother never smoked cigarettes regularly or at all”. A separate question was asked regarding father’s smoking status. Lifetime e-cigarette use was categorized into “ever used” and “never used” using the question “Have you ever used an electronic cigarette (“e-cigarette”)?”

Perceptions of harm of various substances were assessed by asking participants to rate on an eight point Likert scale (1 = very safe and 8 = very dangerous) “How dangerous or safe do you think each of the following drugs or products are?” Respondents were asked to rate perceived harm of flavored e-cigarettes, non-flavored e-cigarettes, tobacco cigarettes, alcohol, and marijuana. Similarly, perceptions of addictiveness were assessed for the same substances using a Likert scale ranging from 1 (not addictive) to 8 (very addictive).

Attitudes regarding acceptability of e-cigarette use in locations where tobacco smoking is banned was assessed by asking, “Do you believe it is OK for someone to use an e-cigarette where regular tobacco smoking is banned?” This question was asked for the following locations: in a bar, at work, in a dormitory room, in a restaurant or store, and in a classroom. Possible responses included “Yes”, “No”, and “Don’t know/not sure”.

Exposure to e-cigarette advertising was assessed by asking respondents to indicate where they had seen e-cigarette advertisements. Respondents were asked to check all applicable responses, which included “On the side of an automobile, bus or other vehicle”, “Billboard”, “Event (e.g. fair, concert, sporting event)”, “Inside a retail store”, “the Internet”, “Magazine or newspaper”, and “Television or movie”.

### Statistical analyses

Data were analyzed using SAS 9.3 [13]. Respondents who indicated their mother was a former or current smoker were reclassified as having a positive smoking status for their mother. Prior evidence demonstrates maternal smoking status is a stronger predictor of tobacco behavior than paternal smoking status [14]. Similarly, in our analysis maternal smoking status was a much stronger predictor of outcomes than paternal smoking status, therefore the remainder of the analyses focus on maternal smoking status. Race was dichotomized into “white” and “non-white”. Due to the low response rate and potential for skewed data due to non-response bias, the data was weighted using population-based weighting [15]. Because limited information was known about both the population and survey respondents, weighting classes were created using the available data which included gender, race, and graduate/undergraduate status. Weights for each class were created using the inverse probability of response for each group. Bivariate analyses were performed to examine associations between participant and outcome variables. Analyses included two-way tests for perceptions of harm and addictiveness and Chi-squared test of association for attitudes of acceptability of e-cigarette use. Because little difference in results existed between flavored and unflavored e-cigarettes all analyses were conducted using responses for flavored e-cigarettes. To correct for multiple comparisons among bivariate analyses, Bonferonni corrections were applied.

Multinomial logistic analysis was conducted using a cumulative logit model for the ordinal variables harm and addictiveness. For acceptability models a dichotomous logistic regression was performed with “no” as the reference, and “I don’t know” excluded. Dichotomous predictors included: gender, white or non-white, e-cigarette use, ever been a smoker, mother ever been a smoker, advertisement seen on the Internet, advertisement seen on TV or movie, and advertisement seen in a magazine or newspaper.

## Results

### E-cigarette advertising exposure

The most common place students had seen advertisements for e-cigarettes was “Television or movie” (53.2%), followed by the Internet (42.0%), at a retail store (35.7%), and in a magazine or newspaper (34.9%) (Table 1).

**Table 1:** Advertising exposure by route

Route	*N*	(%)
Internet	2511	(42.0)
TV or Movie	3185	(53.2)
Retail Store	2138	(35.7)
Billboard	810	(13.6)
Vehicle	483	(8.1)
Event	686	(11.5)
Magazine or newspaper	2062	(34.9)

### Attitudes regarding acceptability of E-cigarette use

More respondents thought it was acceptable (47.1%) versus not acceptable (43.7%) to use an e-cigarette in a bar where tobacco cigarettes are banned, while for all other locations more participants responded it was not acceptable rather than acceptable to use e-cigarettes (Table 2). Nearly 70% of those surveyed indicated that it was not acceptable to use an e-cigarette in class, making it the most unacceptable location to respondents.

**Table 2:** Perceived acceptability of e-cigarette use where tobacco smoking is banned

Location:	Yes *N* (%)	No *N* (%)	Don’t know *N* (%)
Bar	2791 (47.1)	2589 (43.7)	550 (9.3)
Work	1966 (33.1)	3103 (52.3)	864 (14.6)
Dorm	2491 (42.0)	2986 (50.3)	461 (7.8)
Restaurant or Store	1899 (32.1)	3434 (58.0)	586 (9.9)
In class	1370 (23.1)	4115 (69.4)	444 (7.5)

### Perceptions of harm and addiction

Tobacco Cigarettes were perceived as much more harmful (*t* = 71.5 *p* < 0.0001) and addictive than e-cigarettes (*t* = 54.9 *p* < 0.0001) (Table 3). E-cigarettes were perceived as more harmful (*t* = 9.5 *p* < 0.0001) and addictive (*t* = 24 *p* < 0.0001) than marijuana, and less harmful (*t* = 9.6 *p* < 0.0001) but more addictive (*t* = 4.3 *p* < 0.0001) than alcohol.

**Table 3:** Average score for perception of harm and addictiveness for various substances

Perceptions of harm and addictiveness for	Harm x̄ (SD)	Addictiveness x̄ (SD)
Flavored E-Cigarettes	4.49 (2.12)	5.61 (2.17)
Tobacco Cigarettes	6.15 (1.70)	6.93 (1.58)
Alcohol	4.76 (1.81)	5.49 (2.02)
Marijuana	4.20 (2.41)	4.81 (2.55)

Harm and Addictiveness were measured on an 8-point Likert scale (1–8)

Being male, an e-cigarette user, a current tobacco smoker, and having a mother who smoked tobacco cigarettes were all associated with having lower perceived harm and addictiveness of e-cigarettes. Race (white) was associated only with perceived harm (Table 4). All background measures were significantly related with perception of acceptability of use in bars, stores, at work, in class, in dorm (data displayed only for in bars).

**Table 4:** Univariate associations among participant characteristics and route of advertising exposure with e-cigarette perceived harm, addictiveness, and acceptability of use in bars

			Harm			Addictiveness			Acceptability			
			x	t	*n*	x	t	*n*	yes	no	don’t know	*χ*2
Gender		Male	3.98	14.80**	1796	5.15	12.96**	1780	1308 (57.1%)	798 (38.8%)	184 (8.1%)	152**
		Female	4.81		4127	5.90		4054	1482 (40.6%)	1790 (49.2%)	364 (10%)	
Race		White	4.39	6.91**	5065	5.61	0.01	4982	2280 (48.1%)	2055 (43.4%)	402 (8.5%)	21**
		NonWhite	4.87		858	5.61		852	509 (42.8%)	533 (44.8%)	147 (12.4%)	
e-Cigarette		User	3.24	29.6**	1576	4.60	22.39**	1547	1284 (78.9%)	278 (17.1%)	65 (4%)	913**
		Non User	4.96		4347	5.98		4287	1506 (35%)	2310 (53.7%)	484 (11.3%)	
Smoking History												
	Individual	Yes	3.30	22.46**	1147	4.86	13.17**	1117	930 (78.7%)	200 (17%)	51 (4.3%)	593**
		No	4.79		4776	5.79		4717	1860 (39.2%)	2388 (50.3%)	498 (10.5%)	
	Mother	Yes	4.19	7.63**	1985	5.47	3.34**	1950	1081 (55.6%)	716 (36.7%)	146 (7.5%)	84**
		No	4.64		3936	5.67		3883	1708 (42.9%)	1871 (47%)	403 (10.1%)	
Seen Advertising on												
	Internet	Yes	4.20	9.08**	2470	5.46	4.44**	2464	1395 (40.8%)	922 (36.8%)	186 (7.5%)	130**
		No	4.70		3453	5.72		3424	1395 (40.8%)	1666 (48.7%)	363 (10.6%)	
	TV	Yes	4.36	5.06**	3130	5.53	2.97**	3092	1671 (52.6%)	1269 (34.9%)	239 (7.5%)	88**
		No	4.64		2823	5.70		2742	1119 (40.7%)	1319 (48%)	310 (11.3%)	
	Billboard	Yes	4.25	3.43*	783	5.51	1.37	770	473 (58.6%)	293 (36.3%)	41 (5.1%)	55**
		No	4.53		5140	5.62		5064	2317 (45.3%)	2295 (44.8%)	508 (9.9%)	
	Retail Store	Yes	4.18	8.49**	2149	5.45	4.06**	2124	1195 (56.0%)	790 (37.1%)	146 (6.9%)	110**
		No	4.66		3774	5.69		3710	1595 (42.0%)	1798 (47.4%)	402 (10.6%)	
	Vehicle	Yes	4.34	1.57	461	5.53	0.76	447	276 (57.4%)	176 (36.8%)	27 (5.8%)	24**
		No	4.50		5462	5.61		5387	2514 (46.2%)	2411 (44.3%)	521 (9.6%)	
	Event	Yes	3.94	7.21**	686	5.32	3.64**	678	422 (61.8%)	228 (33.4%)	32 (4.8%)	70**
		No	4.56		5237	5.64		5158	2368 (45.2%)	2360 (45%)	516 (9.9%)	
	Paper	Yes	4.30	4.89**	2063	5.54	1.02	2029	1109 (54%)	805 (39.2%)	140 (6.8%)	66**
		No	4.59		3860	5.64		3805	1681 (43.4%)	1783 (46%)	409 (10.6%)	

*p < 0.05, ** *p* <0.01

Each advertising source, except advertisement seen on vehicles, was significantly associated with lower perceived harm. Having seen an advertisement on the Internet, on television, in a retail store, or at an event (e.g., fair, concert, sporting event) were significantly associated with lower perceived addictiveness of e-cigarettes. All forms of advertising exposure were significantly related with perception of acceptability of use in bars, stores, at work, in class, in dorm (data displayed only for in bars).

### Predictors of perceptions of harm, addictiveness and acceptability of use

Being male (aOR 1.7), white (aOR 1.3), ever having used an e-cigarette (aOR 3.2), ever having been a tobacco smoker (aOR 1.8), having a mother who smoked (aOR 1.2), and having seen an advertisement on the Internet (aOR 1.2) all remained significantly associated with lower perceived harm of e-cigarette use (Table 5). Only being male (aOR 1.6) and lifetime e-cigarette use (aOR 2.8) were associated with significantly lower perceived addictiveness of e-cigarettes (Table 5).

**Table 5:** Logistic regression analyses examining predictors of e-cigarette perceived harm and addictiveness

			Perceived Harm (*n* = 5921)		Perceived Addictiveness (*n* = 5832)	
			aOR (Wald CI)	logit	aOR (Wald CI)	logit
Race		NonWhite	1	ref	1	ref
		White	1.31 (1.17–1.47)	0.2702**	0.93 (0.83-1.05)	−0.0698
Gender		Female	1	ref	1	ref
		Male	1.69 (1.54–1.86)	0.5245**	1.60 (1.45–1.76)	0.4689**
E-Cigarette		Non User	1	ref	1	ref
		User	3.17 (2.80–3.58)	1.1524**	2.77 (2.45–3.13)	1.0169**
Smoking History						
	Individual	Yes	1	ref	1	ref
		No	1.78 (1.56–2.04)	0.5775**	1.08 (0.94–1.23)	0.0757
	Mother	Yes	1	ref	1	ref
		No	1.22 (1.11–1.36)	0.1992**	1.03 (0.93–1.14)	0.0504
Advertising seen on						
	Internet	No	1	ref	1	ref
		Yes	1.19 (1.08–1.31)	0.1775*	1.05 (0.95–1.16)	0.0503
	TV	No	1	ref	1	ref
		Yes	1.06 (0.97–1.16)	0.0582	1.04 (0.95–1.15)	0.0430
	Magazine	No	1	ref	1	ref
		yes	0.96 (0.87–1.06)	−0.0402	0.91 (0.82–1.01)	−0.0912

Race was coded 1 = White, 0 = Non-White; Gender was coded 1 = Male, 0 = Female; E-Cigarette User was coded 1 = User, 0 = Non-User; Individual smoking history was coded 1 = Yes, 0 = No; Mother’s smoking history was coded 1 = Yes, 0 = No; Internet advertising was coded 1 = Yes, 0 = No; TV advertising was coded 1 = Yes, 0 = No; Magazine Advertising was coded 1 = Yes, 0 = No

Higher aOR represent lower perceived harm and addictiveness relative to reference group

*Abbreviations*: *aOR* adjusted odds ratio, *CI* confidence interval

*p < 0.05, ** *p* <0.01

*P* values are within model

All factors, except being white and having seen an advertisement in a magazine, were significantly associated with the likelihood of believing e-cigarette use in a bar was acceptable (Table 6). The factors that were significant for believing it was acceptable to use an e-cigarette in a bar were also significant predictors of acceptability of use in other locations with only one exception. Race was a significant predictor of perceiving e-cigarette use in class acceptable, with Whites 1.4 times more likely to perceive it as acceptable than Non-whites.

**Table 6:** Logistic regression analyses predicting perceptions of acceptability of e-cigarette use in various locations

		Acceptable to use in bar (*n* = 4793)		Acceptable to use in store (*n* = 4784)		Acceptable to use at work (*n* = 4792)		Acceptable to use in class (*n* = 4792)		Acceptable to use in dorm (*n* = 4799)	
		AOR (Wald’s CI)	Logit	AOR (Wald’s CI)	Logit	AOR (Wald’s CI)	Logit	AOR (Wald’s CI)	Logit	AOR (Wald’s CI)	Logit
Race	Non-White	1	Ref	1	Ref	1	Ref	1	Ref	1	ref
	White	0.89 (0.75–1.04)	−0.1227	1.02 (0.86–1.22)	0.0222	0.95 (0.80–1.12)	−0.0563	1.44 (1.18–1.77)	0.3675*	1.11 (0.94–1.32)	0.1056
Gender	Female	1	Ref	1	Ref	1	Ref	1	Ref	1	Ref
	Male	1.58 (1.38–1.81)	0.4573**	1.66 (1.44–1.90)	0.5046**	1.75 (1.53–2.01)	0.5608**	1.56 (1.34–1.81)	0.4443**	1.61 (1.40–1.85)	0.4763**
E-Cigarette	Non-User	1	Ref	1	Ref	1	Ref	1	Ref	1	Ref
	User	5.00 (4.22–5.92)	1.6099**	4.45 (3.80–5.21)	1.4920**	4.33 (3.70–5.06)	1.4643**	4.27 (3.62–5.02)	1.4504**	5.94 (5.02–7.02)	1.7810**
Smoking History											
Individual	No	1	Ref	1	Ref	1	Ref	1	Ref	1	Ref
	Yes	2.59 (2.13–3.16)	0.9510**	2.64 (2.21–3.16)	0.9718**	2.47 (2.06–2.95)	0.9023**	2.59 (2.17–3.09)	0.9509**	2.81 (2.31–3.40)	1.0315**
Mother	No	1	Ref	1	Ref	1	Ref	1	Ref	1	Ref
	Yes	1.39 (1.21–1.59)	0.3256**	1.44 (1.24–1.66)	0.3612**	1.45 (1.26–1.67)	0.3735**	1.28 (1.10–1.49))	0.2456*	1.44 (1.25–1.66)	0.3658**
Advertising seen on											
Internet	No	1	Ref	1	Ref	1	Ref	1	Ref	1	Ref
	Yes	1.33 (1.16–1.53)	0.2863**	1.20 (1.04–1.39)	0.1856*	1.16 (1.00–1.33)	0.1439*	1.25 (1.07–1.46)	0.2254*	1.35 (1.17–1.55)	0.2961**
TV	No	1	Ref	1	Ref	1	Ref	1	Ref	1	Ref
	Yes	1.37 (1.20–1.57)	0.3180**	1.33 (1.15–1.53)	0.2843**	1.23 (1.07–1.41)	0.2078*	1.25 (1.07–1.45)	0.2210*	1.33 (1.15–1.52)	0.2817**
Magazine	No	1	Ref	1	Ref	1	Ref	1	Ref	1	Ref
	Yes	1.05 (0.91–1.21)	0.0462	0.99 (0.85–1.15)	−0.0126	1.06 (0.92–1023)	0.0620	0.96 (0.82–1.13)	−0.0383	1.06 (0.91–1.22)	0.0538

Acceptability to use in a location was coded 1 = yes, 0 = no; Race was coded 1 = White, 0 = Non-White; Gender was coded 1 = Male, 0 = Female; E-Cigarette User was coded 1 = User, 0 = Non-User; Individual smoking history was coded 1 = Yes, 0 = No; Mother’s smoking history was coded 1 = Yes, 0 = No; Internet advertising was coded 1 = Yes, 0 = No; TV advertising was coded 1 = Yes, 0 = No; Magazine Advertising was coded 1 = Yes, 0 = No

Higher aOR represent increased perceptions of acceptability of use relative to reference group

*p < 0.05, **p < 0.001

*p*-values are within model

*Abbreviations*: *AOR* adjusted odds ratio, *CI* confidence interval

## Discussion

The results of this study suggest that e-cigarette advertising is associated with perceptions of harm and acceptability of e-cigarette use in places where tobacco cigarettes are banned. Our findings suggest that the substitution of traditional cigarettes by electronic cigarettes as the primary form of nicotine consumption among high school students may be paralleled in college students as we found more students had tried e-cigarettes than were current or former smokers [3]. Traditional cigarette use is still on the decline; however, the increase in e-cigarette use by adolescents and young adults, coupled with the risk of e-cigarettes providing a potential pathway to tobacco cigarettes, threatens to reverse this trend [16]. Further, given that substances such as some flavorings which are common in e-juice may be linked to respiratory disease, e-cigarette use among individuals ages 18–24 is a crucial public health issue [7].

While race was a significant factor in perception of harm, with whites viewing e-cigarettes as less harmful than non-whites, race did not predict perceptions of addictiveness or attitudes about the acceptability of e-cigarette use most locations, with the exception of classrooms. Previous studies have shown that whites are more likely to see e-cigarettes as less harmful than African Americans and Hispanics [17]. Little research exists examining the relationship between ethnicity/race and perceptions of e-cigarette addictiveness. However, in their study of racial differences of cigarette use and beliefs in adolescents, Ma et al. failed to find significant racial differences in perception of ability to quit tobacco, which is an indicator for perceived addictiveness [18].

### E-cigarette advertising exposure

Our findings on advertising exposure suggest that the e-cigarette industry’s targeted advertising towards young people has been successful; more than half our respondents, who are all young adults, have seen an advertisement for e-cigarettes on TV or at the movies. Our findings also demonstrate that having seen ads on the Internet, but not on TV or in magazines, is a predictor of lower perceived harm of e-cigarettes. Current restrictions prevent e-cigarette companies from making health claims in TV or magazine ads. While e-cigarette ads on the Internet are also restricted from making health claims, e-cigarette sellers avoid legal repercussions by using third parties to market e-cigarettes online as having benefits, including aiding in smoking cessation [10]. Consequently, the current research has implications for policy regarding regulation and oversight of Internet-based e-cigarette ads.

Our study also found that advertising exposure through both TV and the Internet was indicative of believing e-cigarette use was acceptable multiple locations. While our cross-sectional design cannot determine causality, previous research has shown that among adolescents, exposure to ads, even those without health claims, increases positive perceptions of e-cigarettes [12]. Further research should explore whether general advertising exposure is a stronger predictor of perceived acceptability than the content of the ad. Currently e-cigarettes are exempt from FDA pre-market reviews, are legal to sell over the internet, require no warning labels, and if e-cigarette advertisements do not make health claims, they are subject to little regulation. However, Congress and the FDA are still determining their policy toward e-cigarettes and results of the growing body of literature on the effects of e-cigarette advertisements may inform policy.

### Attitudes regarding acceptability of E-cigarette use

The current research also reveals that approximately half of the young adults surveyed believed it was permissible to use an e-cigarette in a bar where traditional tobacco cigarettes are banned and more than 30% thought using an e-cigarette in a restaurant was acceptable. In contrast, a 2008 survey of college students found a large majority of respondents favored traditional tobacco cigarettes bans in restaurants (87%) and bars (78%) [19]. Given the rising rates of e-cigarette use, further research should explore the effect of e-cigarette use on perceptions toward tobacco and e-cigarette bans.

### Perceptions of harm and acceptability of use

Our finding that college students perceived e-cigarettes as being slightly more harmful than marijuana but much less harmful than traditional tobacco cigarettes is consistent with previous studies [20]. Our results demonstrated college students perceived e-cigarettes as much more addictive than marijuana, whereas Berg and colleagues found marijuana was perceived as slightly more addictive [20]. This could reflect regional attitudes as their study was performed in the Southeast United States, while the current study was performed in the Midwest.

That college students perceive e-cigarettes as less addictive than traditional tobacco cigarettes is concerning because, among adolescents, intention to use cigarettes has been found to be highly correlated with perceived addictiveness [21]. Given this, adolescents who never would have tried traditional cigarettes may use e-cigarettes.

The strongest predictor of perceived addictiveness, perceived harm, and opinions about acceptability of e-cigarettes use in a different location was participant reported e-cigarette use. Unfortunately, because this is a cross-sectional study, we cannot determine whether beliefs about e-cigarettes as less risky and more acceptable influences use, if use impacts these beliefs, if there is a confounding factor we are not measuring, or some combination of the three.

### Limitations

The current research is not without limitations. First, this study was cross-sectional and therefore it can only reveal association and not causation. For example, it is possible that people who are more likely to believe e-cigarettes are less addictive are also more likely to view e-cigarette ads. Furthermore, the study was not able to control for exposure to e-cigarette advertising. For instance, respondents were asked to indicate which, if any, types of e-cigarette advertising they had ever seen. There was no attempt to capture frequency or duration of exposure for the various types of e-cigarette advertising possible. Despite the utilization of strategies to increase response rate through reminders and incentives, the current study achieved a relatively low response rate of 27%, and thus could have resulted in biased results. However, research exists that challenges the belief that low response rate is directly associated with biased study results [22–24]. Support for the effectiveness of online surveys as a means to collect valid data is mixed [25, 26]. The current study utilized several strategies to increase response rate and thus reduce non-response bias, including the use of a distribution list comprised of currently enrolled students, incentivizing participation through the use of a lottery that included several relatively valuable (e.g., 15-$50) prizes, numerous (e.g., 20 gift cards) chances to win [27], and providing follow-up email reminders to those who had not completed the survey. Lastly, our population consisted of students at one Midwestern university and consequently our findings may not generalize to other college populations or young adults as a whole.

## Conclusion

To our knowledge no population-based studies have examined both exposure to e-cigarette advertisements, perceptions of e-cigarettes, and perceived acceptability of e-cigarette use in places where tobacco smoking is banned. This research discovered that even after accounting for gender, race, e-cigarette use status, and smoking status, exposure to Internet advertisements were associated with lower perceptions of harm of e-cigarette use, and both Internet and TV advertisement exposure was associated with higher perceived acceptability of use in multiple locations such as in bars, stores and at work. The rising prevalence of e-cigarette use among adolescents and young adults coupled with the pervasiveness of e-cigarette advertising highlight the need for further exploration of the impact of advertising on young adult perceptions and use of e-cigarettes. This is especially relevant given that while e-cigarette advertising is now subject to the same restrictions as tobacco advertising, regulating the Internet with regard to e-cigarette advertising and exposure, is largely an impossible task, especially given the frequency with which this young population accesses the Internet.

## Abbreviations

aOR: Adjusted odds ratio
CI: Confidence interval
e-cigarettes: Electronic cigarettes
